# Editorial: Applicative and ecological aspects of mycorrhizal symbioses

**DOI:** 10.3389/fpls.2024.1510941

**Published:** 2024-11-07

**Authors:** Sunil Mundra, Mustafa Morsy

**Affiliations:** ^1^ Department of Biology, College of Science, United Arab Emirates University, Al−Ain, United Arab Emirates; ^2^ Khalifa Center for Genetic Engineering and Biotechnology, United Arab Emirates University, Al−Ain, United Arab Emirates; ^3^ Department of Biological and Environmental Sciences, University of West Alabama, Livingston, AL, United States

**Keywords:** arbuscular mycorrhizal fungi, fungal symbiosis, sustainable agriculture, mycorrhiza, plant health and crop protection

Fungal symbioses, particularly mycorrhizal relationships between fungi and plant roots, are critical for ecological dynamics and have significant implications for agriculture and environmental management (Martin and van der Heijden, 2024). Understanding these interactions is essential for recognizing ttheir roles in ecosystems and potential benefits across various biomes (van der Heijden et al., 2008; Mikryukov et al., 2023; Tedersoo et al., 2014; Bahram et al., 2018). Mycorrhizal fungi enhance plants’ ability to absorb essential nutrients, particularly phosphorus, nitrogen, and micronutrients (Van Der Heijden, 2004). By forming extensive hyphal networks, these fungi increase the surface area for nutrient absorption, improving plant health and growth (Jastrow et al., 1998).

Mycorrhizal fungi also play a vital role in carbon cycling, storing carbon in the soil, influencing global carbon dynamics and helps mitigate climate change (Hawkins et al., 2023). Additionally, they contribute to soil structure by forming aggregates and enhancing porosity, improving water retention and aeration (Jastrow et al., 1998). This ultimately promotes overall soil health and stability. Furthermore, mycorrhizal networks can influence plant competition and coexistence by transferring nutrients between plants and fostering cooperation by forming common mycorrhizal networks (CMNs) where mycobiont establishes an association with the roots of two or more plant species (Henriksson et al., 2023). These symbiotic relationships support plant diversity, enabling various species to thrive in nutrient-poor soils. Many types of mycorrhizal fungi, such as arbuscular mycorrhizal (AM) fungi, ectomycorrhizal (ECM) fungi, orchid mycorrhizal (ORM) fungi, and ericoid mycorrhiza, work with different plant species. These fungi make ecosystems more resilient and help plants survive in harsh conditions like drought, high salt levels, and heavy metal pollution. AM fungi form symbiotic relationships with the roots of most land plants, including many important crops and plants (Brundrett and Tedersoo, 2018). These fungi enhance nutrient uptake, especially phosphorus, and improve plant growth, yield, and stress tolerance, thereby contributing to sustainable agriculture. In this context, nine studies in this Research Topic highlight various aspects of AM fungi in agricultural systems, providing valuable insights into their diversity, identification methods, and potential applications. **Figure 1** summarizes the different benefits AM fungi provide plants, soil, and ecosystems.

**Figure 1:**
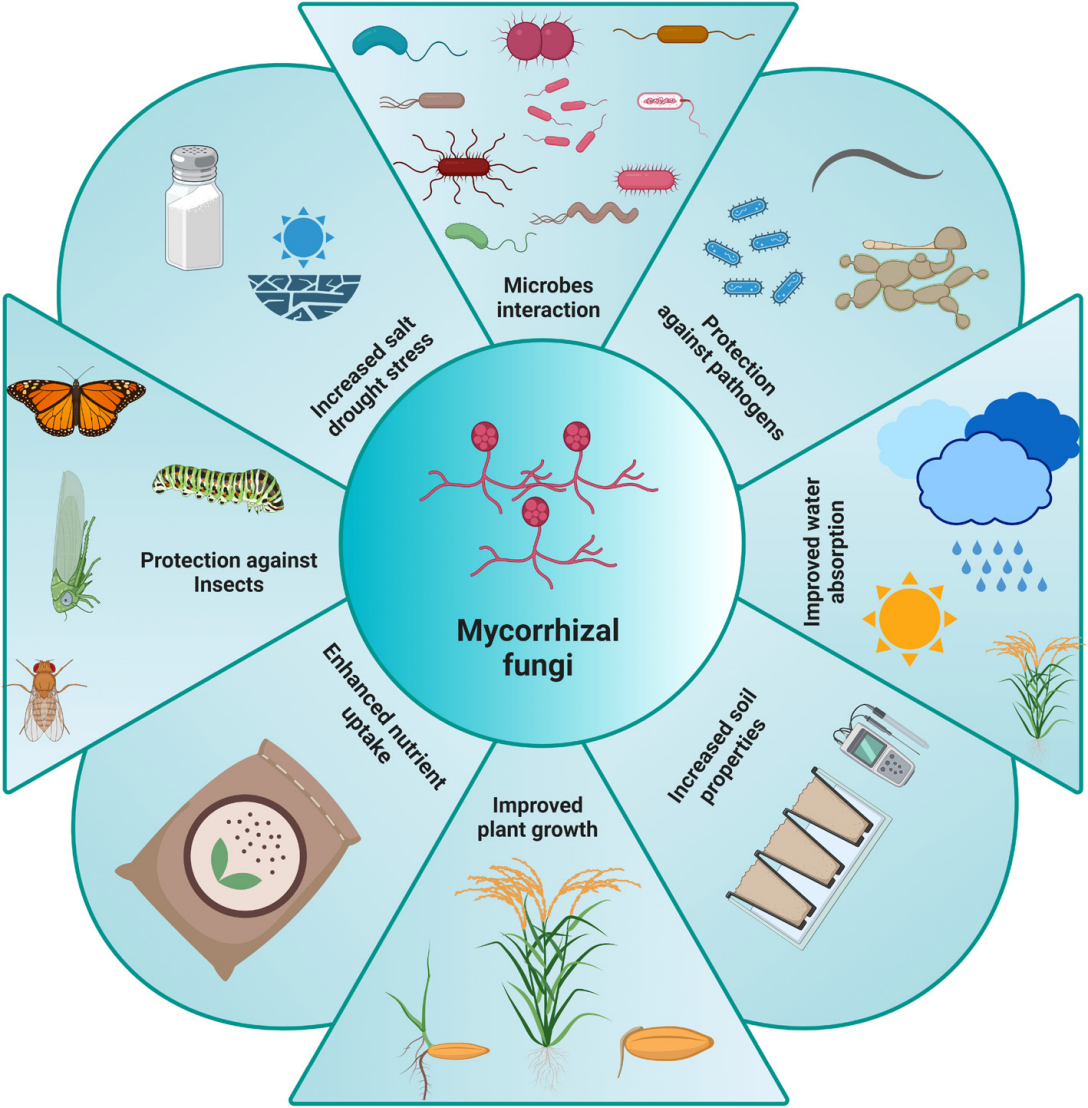
The presence of mycorrhizal fungi provides many benefits to the ecosystem, soil and plants. Understanding their role can improve future farming practices.

This editorial emphasizes the urgency of understanding mycorrhizal symbioses for ecosystem function and agricultural sustainability. The studies showcase the significant roles of mycorrhizal fungi and beneficial microorganisms in enhancing plant health, growth, and resilience while promoting sustainable agricultural approaches. For example, Liang et al. studied the mycorrhizal fungi communities that are connected to *Bulbophyllum tianguii*. They found that the diversity of the fungi was very different between the roots, rhizomes, and rhizosphere soil, with *Sebacina* and *Exophiala* being the most common species. This knowledge aids in the conservation strategies for orchids.

Wang et al. examined how AM fungi influence the growth and herbivore resistance of *Artemisia ordosica* under varying water and nutrient levels. Contrary to expectations, they observed reduced plant height and biomass under low water and nutrient conditions. They reported that while AM fungi generally support plant growth, their effects can be context-dependent, sometimes inhibiting growth in low-resource conditions. Chen et al. studied how adding AMF fungi to *Catalpa bungei* seedlings affected their growth and nitrogen metabolism at different nitrogen levels. They found that adding AM fungi increased the plants’ ability to take in nitrogen and phosphorus and improved photosynthesis at low to medium nitrogen levels. At medium nitrogen levels, AM fungi significantly promoted root growth by altering root hormone levels and improving root architecture and activity. Their findings highlight the potential of AM fungi in tree cultivation strategies. Pokluda et al. investigated the impact of AM fungi and plant growth-promoting microorganisms on onion seedlings. The results showed that the microbial groups worked together to help the plants grow and deal with stress better, especially in organic-rich substrates. This shows how the groups of microbes work together to make things better. Xia et al., investigated the impact of AM fungi inoculation on alfalfa growth and photosynthetic performance under different phosphorus application levels. Using a controlled pot experiment, they demonstrated that inoculation with AM fungi, particularly a mixed inoculation of *Funneliformis mosseae* and *Glomus etunicatum*, significantly improved alfalfa’s photosynthetic efficiency, chlorophyll content and dry matter yield, particularly at optimal phosphorus fertilization. Yu et al., conducted a proteomic analysis of *Poncirus trifoliata* (a common citrus rootstock) roots that were colonized by *Rhizophagus irregularis* and identified key genes and proteins involved in AM colonization. This study contributes to a deeper understanding of the molecular mechanisms underlying AM symbiosis in woody plants like citrus.

Duan et al., discovered that intercropping sugarcane with *Dictyophora indusiata* and *Bacillus aryabhattai* promotes growth through a unique “white root” phenotype and enhanced flavonoid metabolism, providing new strategies for improving sugarcane yield. Maússe-Sitoe and Dames, focused on characterizing AM fungal species associated with maize using single-spore propagation techniques. Through a combination of morphological and molecular methods, they identified several AM fungal genera, including *Claroideoglomus*, *Funneliformis*, *Gigaspora*, *Paraglomus*, and *Rhizophagus*. Their work highlights the diversity of AM fungi in agricultural soils and provides insights into effective methods for isolating and identifying these important symbionts. They proposed that their isolates might be categorized into effective agents that stimulate maize growth and mycorrhization regardless of where they were found. Their findings can greatly contribute to crop productivity and sustainable management of the agricultural ecosystem. Finally, Hassan et al. showed that inoculating *Eruca sativa* with plant growth-promoting bacteria *Jeotgalicoccus* sp. significantly boosts its nutritional quality and biological value by enhancing amino acid and phenolic metabolism, thereby boosting the health benefits of this leafy vegetable.

Harnessing mycorrhizal associations can enhance soil carbon sequestration, contributing to climate change mitigation efforts and improving agricultural sustainability (Averill et al., 2022; Baldrian et al., 2023). Mycorrhizal inoculants can boost crop yields and reduce chemical fertilizer dependence, particularly in organic farming, where soil health and sustainability are prioritized (Martin and van der Heijden, 2024). Moreover, mycorrhizal fungi are valuable in mining and industrial land reclamation, supporting vegetation establishment and promoting ecological recovery. Mycorrhizal fungi are employed in restoration projects to rehabilitate degraded ecosystems. By promoting plant establishment and growth, they aid in restoring biodiversity and ecosystem functionality (Delgado-Baquerizo et al., 2016). They also play a crucial role in restoring degraded ecosystems and enhancing forest resilience.

## Conclusion

Mycorrhizal symbioses provide a complex interplay of ecological benefits and practical applications. Their roles in nutrient cycling, soil health, and plant resilience underscore their importance in natural ecosystems. Furthermore, the potential for enhancing agricultural productivity and supporting ecological restoration positions mycorrhizal fungi as key players in sustainable land management practices. Understanding and harnessing these relationships will be crucial in addressing global challenges in agriculture, conservation, and climate change adaptation.
